# Supplementary material for: Complete mitochondrial genomes of three vulnerable cave bat species and their phylogenetic relationships within the order Chiroptera
Source: PLoS One. 2024 Aug 22;19(8):e0308741. doi: 10.1371/journal.pone.0308741 (PMC11340975; doi:10.1371/journal.pone.0308741)
Supplement: S1 Table — (DOCX) [file pone.0308741.s004.docx]

**S1 Table. Chiroptera species used in the phylogenetic reconstructions with whole mitogenome sequences indicating their respective family affiliations and GenBank accession numbers.**

| **Family** | **Species** | **GenBank accession** |
| --- | --- | --- |
| Emballonuridae | *Saccopteryx leptura* | KY681816 |
| Emballonuridae | *Taphozous melanopogon* | MZ286363 |
| Furipteridae | *Furipterus horrens* | MK033190 |
| Hipposideridae | *Rhinolophus affinis* | MT845219 |
| Megadermatidae | *Macroderma gigas* | MW006543 |
| Molossidae | *Tadarida teniotis* | KY581661 |
| Mormoopidae | *Pteronotus personatus* | KU569221 |
| Mystacinidae | *Mystacina tuberculata* | AY960981 |
| Natalidae | *Natalus macrourus* | OR879255 |
| Noctilionidae | *Noctilio leporinus* | KU743910 |
| Phyllostomidae | *Artibeus lituratus* | JN209840 |
| Phyllostomidae | *Chrotopterus auritus* | KU743905 |
| Phyllostomidae | *Diaemus youngi* | KU743906 |
| Phyllostomidae | *Diphylla ecaudata* | KU743911 |
| Phyllostomidae | *Ectophylla alba* | MH260572 |
| Phyllostomidae | *Glyphonycteris daviesi* | KU743912 |
| Phyllostomidae | *Hsunycteris thomasi* | KU743907 |
| Phyllostomidae | *Lonchorhina aurita* | OR879254 |
| Phyllostomidae | *Macrotus californicus* | KU743909 |
| Phyllostomidae | *Tonatia bidens* | MZ391834 |
| Pteropodidae | *Balionycteris maculata* | OK274257 |
| Pteropodidae | *Casinycteris argynnis* | MN816300 |
| Pteropodidae | *Cynopterus sphinx* | MN816305 |
| Pteropodidae | *Eidolon helvum* | MN816307 |
| Pteropodidae | *Eonycteris spelaea* | MN816308 |
| Pteropodidae | *Epomophorus crypturus* | MN816310 |
| Pteropodidae | *Epomops buettikoferi* | MN816323 |
| Pteropodidae | *Hypsignathus monstrosus* | MN816326 |
| Pteropodidae | *Lissonycteris angolensis* | MN816334 |
| Pteropodidae | *Macroglossus sobrinus* | MN816327 |
| Pteropodidae | *Megaerops niphanae* | MN816328 |
| Pteropodidae | *Megaloglossus azagnyi* | MN816329 |
| Pteropodidae | *Micropteropus pusillus* | MN816332 |
| Pteropodidae | *Myonycteris relicta* | MN816338 |
| Pteropodidae | *Nanonycteris veldkampii* | MN816340 |
| Pteropodidae | *Nyctimene cephalotes* | MN816342 |
| Pteropodidae | *Plerotes anchietae* | MN816343 |
| Pteropodidae | *Pteropus vampyrus* | KP214033 |
| Pteropodidae | *Rousettus obliviosus* | MN816358 |
| Pteropodidae | *Scotonycteris zenkeri* | MN816363 |
| Pteropodidae | *Sphaerias blanfordi* | MN816364 |
| Rhinolophidae | *Hipposideros larvatus* | MN056567 |
| Vespertilionidae | *Corynorhinus rafinesquii* | JN209841 |
| Vespertilionidae | *Eudiscopus denticulus* | MW085031 |
| Vespertilionidae | *Hypsugo alaschanicus* | KT380130 |
| Vespertilionidae | *Ia io* | MZ579648 |
| Vespertilionidae | *Kerivoula minuta* | OK274260 |
| Vespertilionidae | *Lasionycteris noctivagans* | MT774149 |
| Vespertilionidae | *Lasiurus borealis* | JN209842 |
| Vespertilionidae | *Miniopterus fuliginosus* | MH523628 |
| Vespertilionidae | *Murina leucogaster* | KM893454 |
| Vespertilionidae | *Myotis albescens* | MF143497 |
| Vespertilionidae | *Nyctalus aviator* | MK167360 |
| Vespertilionidae | *Pipistrellus kuhlii* | KU058655 |
| Vespertilionidae | *Plecotus auritus* | HM164052 |
| Vespertilionidae | *Tylonycteris fulvida* | MZ457524 |
| Vespertilionidae | *Vespertilio sinensis* | KJ081440 |
