# Supplementary material for: Complete mitochondrial genomes of three vulnerable cave bat species and their phylogenetic relationships within the order Chiroptera
Source: PLoS One. 2024 Aug 22;19(8):e0308741. doi: 10.1371/journal.pone.0308741 (PMC11340975; doi:10.1371/journal.pone.0308741)
Supplement: S2 Table — (DOCX) [file pone.0308741.s005.docx]

**S2 Table. Comparison of the length of the four stems that conform the secondary structure of the tRNAs of *Furipterus horrens*, *Lonchorhina aurita*, and *Natalus macrourus*.**

| **tRNA** | **Aceptor stem length (bp)** | | | **Anticodon stem length (bp)** | | | **TψC stem length (bp)** | | | **DHU stem length (bp)** | | |
| --- | --- | --- | --- | --- | --- | --- | --- | --- | --- | --- | --- | --- |
|  | *F. horrens* | *L. aurita* | *N. macrourus* | *F. horrens* | *L. aurita* | *N. macrourus* | *F. horrens* | *L. aurita* | *N. macrourus* | *F. horrens* | *L. aurita* | *N. macrourus* |
| trnF (gaa) | 7 | 7 | 7 | 5 | 5 | 5 | 5 | 4 | 5 | 4 | 4 | 4 |
| trnV (tac) | 7 | 7 | 7 | 5 | 5 | 5 | 4 | 3 | 4 | 4 | 4 | 4 |
| trnL2 (taa) | 7 | 7 | 7 | 4 | 4 | 4 | 5 | 5 | 5 | 4 | 4 | 4 |
| trnI (gat) | 7 | 7 | 7 | 5 | 5 | 5 | 5 | 5 | 5 | 3 | 3 | 3 |
| trnQ (ttg) | 7 | 7 | 7 | 4 | 4 | 4 | 4 | 4 | 5 | 4 | 4 | 4 |
| trnM (cat) | 7 | 7 | 7 | 5 | 5 | 5 | 5 | 5 | 5 | 4 | 4 | 4 |
| trnW (tca) | 7 | 7 | 7 | 5 | 5 | 5 | 5 | 5 | 5 | 4 | 4 | 4 |
| trnA (tgc) | 7 | 7 | 7 | 5 | 5 | 5 | 5 | 5 | 5 | 4 | 4 | 4 |
| trnN (gtt) | 7 | 7 | 7 | 5 | 5 | 5 | 5 | 5 | 5 | 3 | 3 | 3 |
| trnC (gca) | 7 | 7 | 7 | 5 | 5 | 5 | 4 | 4 | 4 | 4 | 4 | 4 |
| trnY (gta) | 7 | 7 | 7 | 5 | 5 | 5 | 5 | 5 | 5 | 3 | 3 | 3 |
| trnS2 (tga) | 7 | 7 | 7 | 5 | 5 | 5 | 5 | 5 | 5 | 3 | 3 | 3 |
| trnD (gtc) | 7 | 7 | 7 | 5 | 5 | 5 | 5 | 5 | 5 | 4 | 4 | 4 |
| trnK (ttt) | 7 | 7 | 7 | 5 | 5 | 5 | 5 | 5 | 5 | 4 | 4 | 4 |
| trnG (tcc) | 7 | 7 | 7 | 5 | 5 | 5 | 5 | 5 | 5 | 4 | 4 | 4 |
| trnR (tcg) | 7 | 6 | 7 | 5 | 5 | 5 | 4 | 5 | 5 | 4 | 4 | 4 |
| trnH (gtg) | 7 | 7 | 7 | 5 | 5 | 5 | 5 | 3 | 4 | 4 | 4 | 4 |
| trnS1 (gct) | 7 | 7 | 7 | 4 | 3 | 3 | 5 | 5 | 5 | - | - | - |
| trnL1 (tag) | 7 | 7 | 7 | 5 | 5 | 5 | 4 | 4 | 4 | 4 | 4 | 4 |
| trnE (ttc) | 7 | 7 | 7 | 5 | 5 | 5 | 5 | 5 | 5 | 4 | 4 | 4 |
| trnT (tgt) | 7 | 7 | 7 | 5 | 5 | 5 | 4 | 4 | 5 | 3 | 3 | 3 |
| trnP (tgg) | 7 | 7 | 7 | 5 | 5 | 5 | 5 | 5 | 5 | 4 | 4 | 4 |
