# Supplementary material for: Complete mitochondrial genomes of three vulnerable cave bat species and their phylogenetic relationships within the order Chiroptera
Source: PLoS One. 2024 Aug 22;19(8):e0308741. doi: 10.1371/journal.pone.0308741 (PMC11340975; doi:10.1371/journal.pone.0308741)
Supplement: S3 Table — (DOCX) [file pone.0308741.s006.docx]

**S3 Table. Microsatellites sequences found in the CR of the mitochondrial genome of *Furipterus horrens*.**

| **Position** | **Microsatellite** | **Number of repeats** |
| --- | --- | --- |
| 25 | TAT | 3 |
| 405 | TT | 3 |
| 628 | TA | 3 |
| 636 | CC | 3 |
| 651 | ACGTAC | 14 |
| 736 | CGCACA | 5 |
| 767 | GTACAC | 11 |
| 843 | TA | 3 |
