# Supplementary material for: Complete mitochondrial genomes of three vulnerable cave bat species and their phylogenetic relationships within the order Chiroptera
Source: PLoS One. 2024 Aug 22;19(8):e0308741. doi: 10.1371/journal.pone.0308741 (PMC11340975; doi:10.1371/journal.pone.0308741)
Supplement: S4 Table — (DOCX) [file pone.0308741.s007.docx]

**S4 Table.** **Microsatellites sequences found in the CR of the mitochondrial genome of *Lonchorhina aurita*.**

| **Position** | **Microsatellite** | **Number of repeats** |
| --- | --- | --- |
| 53 | AT | 3 |
| 78 | TA | 3 |
| 119 | TA | 3 |
| 213 | AT | 3 |
| 531 | TT | 4 |
| 699 | TG | 3 |
| 807 | CA | 3 |
| 813 | CGTACA | 18 |
| 922 | GCACAC | 3 |
| 949 | CGTACA | 3 |
| 987 | CC | 3 |
| 995 | CC | 3 |
| 1017 | AT | 3 |
