# Supplementary material for: Complete mitochondrial genomes of three vulnerable cave bat species and their phylogenetic relationships within the order Chiroptera
Source: PLoS One. 2024 Aug 22;19(8):e0308741. doi: 10.1371/journal.pone.0308741 (PMC11340975; doi:10.1371/journal.pone.0308741)
Supplement: S5 Table — (DOCX) [file pone.0308741.s008.docx]

**S5 Table. Microsatellites sequences found in the CR of the mitochondrial genome of *Natalus macrourus*.**

| **Position** | **Microsatellite** | **Number of repeats** |
| --- | --- | --- |
| 65 | TA | 3 |
| 394 | TTT | 3 |
| 454 | ATA | 3 |
| 624 | ATA | 3 |
| 631 | CC | 5 |
| 653 | CGTACA | 37 |
| 904 | CC | 3 |
| 912 | CC | 3 |
| 941 | AT | 4 |
| 1153 | AT | 3 |
