# Supplementary material for: Complete mitochondrial genomes of three vulnerable cave bat species and their phylogenetic relationships within the order Chiroptera
Source: PLoS One. 2024 Aug 22;19(8):e0308741. doi: 10.1371/journal.pone.0308741 (PMC11340975; doi:10.1371/journal.pone.0308741)
Supplement: S1 Fig — (PDF) [file pone.0308741.s001.pdf]

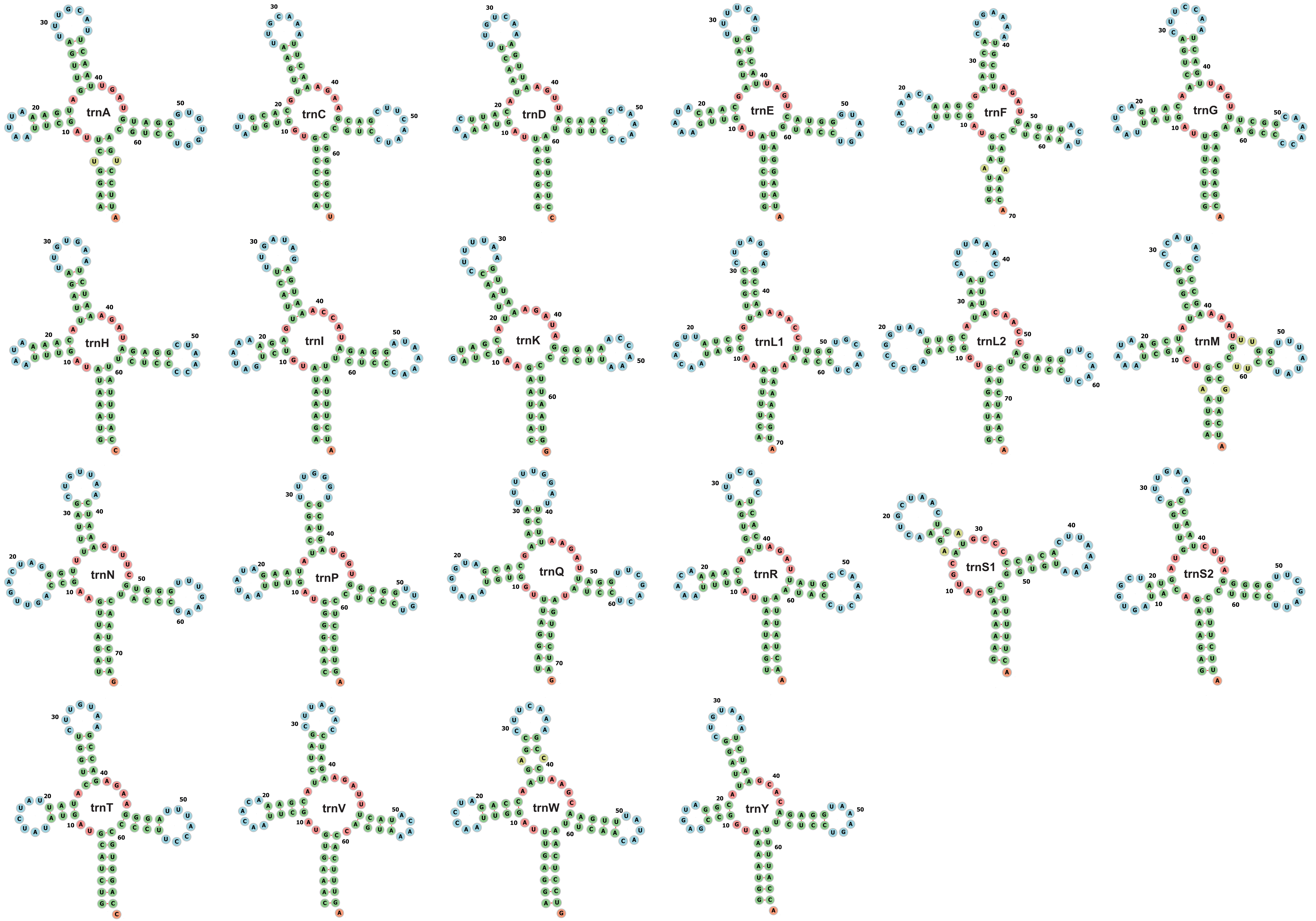
trnA
trnC
trnD
trnE
trnF
trnG
trnH
trnI
trnK
trnL1
trnL2
trnM
trnN
trnP
trnQ
trnR
trnS1
trnS2
trnT
trnV
trnW
trnY
