# Supplementary material for: Complete mitochondrial genomes of three vulnerable cave bat species and their phylogenetic relationships within the order Chiroptera
Source: PLoS One. 2024 Aug 22;19(8):e0308741. doi: 10.1371/journal.pone.0308741 (PMC11340975; doi:10.1371/journal.pone.0308741)
Supplement: Attachment — Submitted filename: PONE-D-24-05101.pdf [file pone.0308741.s009.pdf]

PLOS ONE

Complete mitochondrial genome of three vulnerable cave bat species

--Manuscript Draft--

**Manuscript Number:**	PONE-D-24-05101
**Article Type:**	Research Article
**Full Title:**	Complete mitochondrial genome of three vulnerable cave bat species
**Short Title:**	Mitogenomes of three cave bats
**Corresponding Author:**	Santelmo Vasconcelos<br>Instituto Tecnológico Vale<br>Belém, Pará BRAZIL
**Keywords:**	Amazon; Furipterus; Lonchorhina; Natalus; mitogenome; trogloxenes
**Abstract:**	The IUCN Red List of Threatened Species contains 175 Brazilian bat species that are threatened by extinction in some degree. From this perspective, it is essential to expand the knowledge about the genetic diversity of vulnerable bats. Genomic sequencing can be useful to generate robust and informative genetic references increasing resolution when analyzing relationships among populations, species, or higher taxonomic levels. In this study, we sequenced and characterized in detail the first complete mitochondrial genome of Furipterus horrens, Lonchorhina aurita, and Natalus macrourus, and investigated the phylogenetic position of these species based on protein-coding genes (PCGs). The mitogenomes of these species are 16,516, 16,697, and 16,668 bp in length, respectively, and each encodes 13 PCGs, 22 tRNA genes, two rRNA genes, and a putative control region (CR). In the three species, genes were arranged similarly to all other bats with mitogenomes previously described, and nucleotide composition was also consistent with the reported range. The length and arrangement of rrnS and rrnL were consistent with those of other bat species, showing a positive AT-skew and a negative GC-skew. Except for trnS1, for which we did not observe the DHU arm, all other tRNAs showed the cloverleaf secondary structure in the three species. In addition, the mitogenomes showed minor differences in start and stop codons, and in all PCGs, codons ending in adenine were more common compared to those ending in guanine. We found that PCGs of the three species use multiple codons to encode each amino acid, following the previously documented pattern. Furthermore, all PCGs are under purifying selection, with atp8 experiencing the most relaxed purifying selection.
**Order of Authors:**	Michele Molina
	Guilherme Oliveira
	Renato R. M. Oliveira
	Gisele L. Nunes
	Eder S. Pires
	Xavier Prous
	Mariane Ribeiro
	Santelmo Vasconcelos
**Additional Information:**	
**Question**	**Response**
**Financial Disclosure**<br><br>Enter a financial disclosure statement that describes the sources of funding for the work included in this submission. Review the submission guidelines for detailed requirements. View published research	Yes

Powered by Editorial Manager® and ProduXion Manager® from Aries Systems Corporation

articles from *PLOS ONE* for specific examples.<br><br>This statement is required for submission and **will appear in the published article** if the submission is accepted. Please make sure it is accurate.<br><br>**Funded studies**<br>Enter a statement with the following details:<br>• Initials of the authors who received each award<br>• Grant numbers awarded to each author<br>• The full name of each funder<br>• URL of each funder website<br>• Did the sponsors or funders play any role in the study design, data collection and analysis, decision to publish, or preparation of the manuscript?<br><br>Did you receive funding for this work?	
Please add funding details.<br>as follow-up to "**Financial Disclosure**<br><br>Enter a financial disclosure statement that describes the sources of funding for the work included in this submission. Review the submission guidelines for detailed requirements. View published research articles from *PLOS ONE* for specific examples.<br><br>This statement is required for submission and **will appear in the published article** if the submission is accepted. Please make sure it is accurate.	This work was funded by Vale (Projeto Diversidade Biológica de Cavernas, R100603.CD.0X). Guilherme Oliveira is a CNPq (Conselho Nacional de Desenvolvimento Científico) fellow (307479/2016-1).

**Funded studies**<br>Enter a statement with the following details:<br>• Initials of the authors who received each award<br>• Grant numbers awarded to each author<br>• The full name of each funder<br>• URL of each funder website<br>• Did the sponsors or funders play any role in the study design, data collection and analysis, decision to publish, or preparation of the manuscript?<br><br>Did you receive funding for this work?"	
Please select the country of your main research funder (please select carefully as in some cases this is used in fee calculation).<br>as follow-up to "**Financial Disclosure**<br><br>Enter a financial disclosure statement that describes the sources of funding for the work included in this submission. Review the submission guidelines for detailed requirements. View published research articles from *PLOS ONE* for specific examples.<br><br>This statement is required for submission and **will appear in the published article** if the submission is accepted. Please make sure it is accurate.<br><br>**Funded studies**<br>Enter a statement with the following details:<br>• Initials of the authors who received each award<br>• Grant numbers awarded to each author<br>• The full name of each funder<br>• URL of each funder website<br>• Did the sponsors or funders play any role in the study design, data collection and analysis, decision to publish, or preparation of the manuscript?<br><br>Did you receive funding for this work?"	BRAZIL - BR
**Competing Interests**	The authors declare no conflict of interest.

Use the instructions below to enter a competing interest statement for this submission. On behalf of all authors, disclose any competing interests that could be perceived to bias this work—acknowledging all financial support and any other relevant financial or non-financial competing interests.<br><br>This statement is required for submission and **will appear in the published article** if the submission is accepted. Please make sure it is accurate and that any funding sources listed in your Funding Information later in the submission form are also declared in your Financial Disclosure statement.<br><br>View published research articles from *PLOS ONE* for specific examples.<br><br>**NO authors have competing interests**<br><br>Enter: *The authors have declared that no competing interests exist.*<br><br>**Authors with competing interests**<br><br>Enter competing interest details beginning with this statement:<br><br>*I have read the journal's policy and the authors of this manuscript have the following competing interests: [insert competing interests here]*<br><br>* typeset	
**Ethics Statement**<br><br>Enter an ethics statement for this submission. This statement is required if the study involved:<br><br>• Human participants<br>• Human specimens or tissue<br>• Vertebrate animals or cephalopods<br>• Vertebrate embryos or tissues	All field activities and tissues sampled for the experiments were in accordance with the sampling permit 49994-12, granted by ICMBio/MMA (Brazilian Ministry of Environment).

Powered by Editorial Manager® and ProduXion Manager® from Aries Systems Corporation

• Field research<br><br>Write "N/A" if the submission does not require an ethics statement.<br><br>General guidance is provided below. Consult the submission guidelines for detailed instructions. **Make sure that all information entered here is included in the Methods section of the manuscript.**	

Powered by Editorial Manager® and ProduXion Manager® from Aries Systems Corporation

**Format for specific study types**<br><br>**Human Subject Research (involving human participants and/or tissue)**<br>• Give the name of the institutional review board or ethics committee that approved the study<br>• Include the approval number and/or a statement indicating approval of this research<br>• Indicate the form of consent obtained (written/oral) or the reason that consent was not obtained (e.g. the data were analyzed anonymously)<br><br>**Animal Research (involving vertebrate animals, embryos or tissues)**<br>• Provide the name of the Institutional Animal Care and Use Committee (IACUC) or other relevant ethics board that reviewed the study protocol, and indicate whether they approved this research or granted a formal waiver of ethical approval<br>• Include an approval number if one was obtained<br>• If the study involved *non-human primates*, add *additional details* about animal welfare and steps taken to ameliorate suffering<br>• If anesthesia, euthanasia, or any kind of animal sacrifice is part of the study, include briefly which substances and/or methods were applied<br><br>**Field Research**<br><br>Include the following details if this study involves the collection of plant, animal, or other materials from a natural setting:<br>• Field permit number<br>• Name of the institution or relevant body that granted permission	
**Data Availability**<br><br>Authors are required to make all data underlying the findings described fully available, without restriction, and from the time of publication. PLOS allows rare exceptions to address legal and ethical concerns. See the PLOS Data Policy and FAQ for detailed information.	Yes - all data are fully available without restriction

Powered by Editorial Manager® and ProduXion Manager® from Aries Systems Corporation

A Data Availability Statement describing where the data can be found is required at submission. Your answers to this question constitute the Data Availability Statement and **will be published in the article**, if accepted.<br><br>**Important:** Stating 'data available on request from the author' is not sufficient. If your data are only available upon request, select 'No' for the first question and explain your exceptional situation in the text box.<br><br>Do the authors confirm that all data underlying the findings described in their manuscript are fully available without restriction?	
**Describe where the data may be found in full sentences. If you are copying our sample text, replace any instances of XXX with the appropriate details.**<br><br>• If the data are **held or will be held in a public repository**, include URLs, accession numbers or DOIs. If this information will only be available after acceptance, indicate this by ticking the box below. For example: *All XXX files are available from the XXX database (accession number(s) XXX, XXX.).*<br>• If the data are all contained **within the manuscript and/or Supporting Information files**, enter the following: *All relevant data are within the manuscript and its Supporting Information files.*<br>• If neither of these applies but you are able to provide **details of access elsewhere**, with or without limitations, please do so. For example:<br><br>*Data cannot be shared publicly because of [XXX]. Data are available from the XXX Institutional Data Access / Ethics Committee (contact via XXX) for researchers who meet the criteria for access to confidential data.*<br><br>*The data underlying the results presented in the study are available from (include the name of the third party*	The three mitochondrial genomes described were deposited in the GenBank database under the accession numbers MK033190, OR879254 and OR879255.

and contact information or URL).<br>• This text is appropriate if the data are owned by a third party and authors do not have permission to share the data.<br><br>* typeset	
Additional data availability information:	

Manuscript file with tables

Click here to access/download;Manuscript;Molina_et_al_ChiropteraMitogen

Complete mitochondrial genome of three vulnerable cave bat species

Michele Molina[1,2]*, Guilherme Oliveira[1,2], Renato R. M. Oliveira[1], Gisele L. Nunes[1], Eder S. Pires[1], Xavier Prous[3], Mariane Ribeiro[3], Santelmo Vasconcelos[1,4]*

[1] Instituto Tecnológico Vale, Rua Boaventura da Silva 955, 66055-090, Belém, Pará, Brazil.

[2] Programa de Pós-Graduação em Genética e Biologia Molecular, Universidade Federal do Pará, Belém, Pará, Brazil.

[3] Gerência de Espeleologia, Vale, Nova Lima, Minas Gerais, Brazil.

[4] Programa de Pós-Graduação em Biodiversidade e Evolução, Museu Paraense Emílio Goeldi, Belém, Pará, Brazil.

* Corresponding authors

michele.molina@pq.itv.org (MM); santelmo.vasconcelos@itv.org (SV)

Abstract

The IUCN Red List of Threatened Species contains 175 Brazilian bat species that are threatened by extinction in some degree. From this perspective, it is essential to expand the knowledge about the genetic diversity of vulnerable bats. Genomic sequencing can be useful to generate robust and informative genetic references increasing resolution when analyzing relationships among populations, species, or higher taxonomic levels. In this study, we sequenced and characterized in detail the first complete mitochondrial genome of *Furipterus horrens*, *Lonchorhina aurita*, and *Natalus macrourus*, and investigated the phylogenetic position of these species based on protein-coding genes (PCGs). The mitogenomes of these species are 16,516, 16,697, and 16,668 bp in length, respectively, and each encodes 13 PCGs, 22 tRNA genes, two rRNA genes, and a putative control region (CR). In the three species, genes were arranged similarly to all other bats with mitogenomes previously described, and nucleotide composition was also consistent with the reported range. The length and arrangement of rrnS and rrnL were consistent with those of other bat species, showing a positive AT-skew and a negative GC-skew. Except for trnS1, for which we did not observe the DHU arm, all other tRNAs showed the cloverleaf secondary structure in the three species. In addition, the mitogenomes showed minor differences in start and stop codons, and in all PCGs, codons ending in adenine were more common compared to those ending in guanine. We found that PCGs of the three species use multiple codons to encode each amino acid, following the previously documented pattern. Furthermore, all PCGs are under purifying selection, with atp8 experiencing the most relaxed purifying selection.

Keywords: Amazon, *Furipterus*, *Lonchorhina*, Mitogenome, *Natalus*, Trogloxenes

1. Introduction

The order Chiroptera encompasses a wide range of species and wide geographic distribution, reflecting their great functional, morphological, and ecological diversities [1]. Due to this diversity, bats play an essential role in the ecosystem by providing services such as pollination, seed dispersal, insect control, and organic matter supply to subterranean ecosystems, contributing to the energy flow of both hypogean and epigean ecosystems [2-3]. This relationship among bats, karst ecosystems, and their surroundings is important for some species seeking protection against predators and extreme weather conditions in these landscapes, ensuring the maintenance and survival of their populations [4-6].

The Brazilian landscapes are rich in caves, with karst areas accounting for an estimated 7% of the country's surface area [7-8]. As a result, the country hosts 181 of the 1450 known bat species, or about 13% of the global richness of the group [9-13]. It is important to note that almost half (44%) of bat species in Brazil are classified as trogloxenes, i.e., although they primarily live underground, they also emerge from their habitat in search of food [14]. However, there are anthropogenic interventions that potentially damage cave environments, which are related to important economic activities such as mining, agriculture, and tourism [15-18], posing challenges to bat conservation as species are directly affected by habitat disturbance.

Thus, since cave systems rely on the allochthonous supply of resources by bats (guano deposition, food scraps, and eventually their carcasses), and bats need these underground habitats for refuge, the functioning of this delicate ecological system must be ensured. To achieve this goal, it is necessary to preserve and protect not only the underground physical environment but also the surrounding influence area (or foraging

area) and the cave bats themselves. Only by maintaining bats, as energy importers, can meaningful ecological interactions be maintained, leading to the preservation these ecosystems' biodiversity.

However, according to the International Union for Conservation of Nature (IUCN) Red List of Threatened Species, 1,332 Chiroptera species are threatened in some category [19]. About 13% of these species (175 spp.) occur in Brazil, including cave species such as *Furipterus horrens* [20], *Lonchorhina aurita* [21], and *Natalus macrourus* [22] (identified in the original list as *Natalus espiritosantensis* – [23]), which are categorized as vulnerable. *Lonchorhina aurita* remained on the Red List between 2014 and 2022 but was reclassified as Near Threatened in the current version, showing that it still needs attention. These three species are trogloxenes, insectivorous, and occur in the Amazon, being of great importance for the conservation of the karst areas of this important biome.

From this perspective, increasing the knowledge of the fauna of cave bats is essential, especially endangered species. Thus, genome sequencing based on next-generation technologies can be an important tool for analyses that can support important decisions regarding species and conservation of underground ecosystems, such as generating informative genetic references as complete mitochondrial genomes [24-25]. Mitogenomes (mtDNA) are relatively small for animal species, generally ranging in length between 13-22 kbp, and can be obtained from low-coverage sequencing. This approach is highly cost-effective compared to other strategies for characterizing genetic diversity, while significantly increasing resolution when analyzing relationships among populations, species, or higher taxonomic levels [26].

In this study, we generated and assembled the first complete mitogenomes for the threatened cave bats *F. horrens*, *L. aurita*, and *N. macrourus*, assembling and analyzing

in detail the whole sequences of the three species, aiming to increase the genetic data on endangered species in the Brazilian Amazon to support biodiversity conservation strategies in the region. Specifically, we analyzed the nucleotide composition of the entire mtDNA and examined the codon usage profiles and selective constraints of protein-coding genes (PCGs). We also described the secondary structure of each identified tRNA gene and examined the organization of the control region in detail. Finally, we assess the phylogenetic position of these three species among other Chiroptera representatives based on amino acid supermatrix with the 13 PCGs.

2. Materials and methods

2.1. Sampling and DNA sequencing

Fieldwork was carried out in the Carajás National Forest, in the Brazilian state of Pará, Eastern Amazon (Table 1). Tissue samples were collected from dactylopatagium of adult specimens of *F. horrens*, *L. aurita* and *N. macrourus* and preserved in 2 mL cryogenic microtubes containing absolute ethanol and kept refrigerated for subsequent laboratory processing. Total genomic DNA was extracted with the DNeasy Blood & Tissue kit (Qiagen), following the manufacturer's instructions for vertebrate. Then, for mitogenome sequencing, paired-end shotgun libraries were prepared using the Illumina DNA Prep kit (Illumina), according to the manufacturer's instructions, and sequenced on Illumina NextSeq 500 platform (Illumina, San Diego, CA, USA), using the NextSeq 500/550 v2.5 kit (300 cycles, 2× 150 bp).

Table 1. Bats sampling locations across Serra dos Carajás, Pará, Brazil, and complete mitochondrial genome sequences retrieved from NCBI.

Species	voucher	Sample location; Coordinates	GenBank accession
Furipterus horrens	ITV8911	S11D0064; -6,392318; -50,313616	MK033190
Lonchorhina aurita	ITV8916	S11B0094; -6,3349066; -50,393498	OR879254
Natalus macrourus	ITV8904	S11B0094; -6,3349066; -50,393498	OR879255

2.2. Mitochondrial genome assembly, annotation, and analyses

After sequencing, the data were processed using the AdapterRemoval v2 tool [27] with a minimum quality threshold of Phred > 20. Then, the high-quality reads were subjected to a quality check step using the FastQC software (http://bioinformatics.babraham.ac.uk/projects/fastqc/). Circularized mitogenomes were assembled using NOVOPlasty v4.2 [28], with subsequent checking and manual corrections in the software Geneious Prime v2023 (Biomatters). The annotation was performed with the MITOS2 webserver [29], and then gene boundaries were manually checked and curated in Geneious. tRNAs and their secondary structure were identified and predicted with MiTFi [30] in MITOS2, being subsequently depicted with the FORNA webserver [31]. Finally, the control region (CR) was also analyzed in more detail, using the Microsatellite Repeats Finder webserver [32] to detect microsatellite sequences.

2.3. Genetic heterogeneity, and genetic diversity analysis

Nucleotide composition was calculated in Geneious, and AT and GC skew was computed by using the formulas (A% − T%)/(A% + T%) and (G% − C%)/(G% + C%) [33]. Codon usage and relative synonym codon usage values (RSCU) for each PCG were estimated using the vertebrate mitochondrial code in the Codon Usage webserver (http://www.bioinformatics.org/sms2/codon_usage.html).

To explore selective constraints, the number of synonymous and nonsynonymous substitutions per synonymous and nonsynonymous site (Ks = dS = SS/LS, and Ka = dN = SA/LA) were estimated to calculate the Ka/Ks (ω) ratio in the software MEGA v11 using the K2P model [34], based on pairwise comparisons between each one of the three sampled species and a representative mitogenome of all the other Chiroptera families with data available in GenBank (https://www.ncbi.nlm.nih.gov/genbank/): *Hipposideros larvatus* (Hipposideridae; accession number MN056567), *Rhinolophus affinis* (Rhinolophidae; MT845219), *Mystacina tuberculata* (Mystacinidae; AY960981), *Pteronotus personatus* (Mormoopidae; KU569221), *Myotis albescens* (Vespertilionidae; MF143497), *Tadarida teniotis* (Molossidae; KY581661), *Saccopteryx leptura* (Emballonuridae; KY681816), *Miniopterus fuliginosus* (Miniopteridae; MH523628), *Noctilio leporinus* (Noctilionidae; KU743910), *Lissonycteris angolensis* (Pteropodidae; MN816334), and *Macroderma gigas* (Megadermnatidae; MW006543). The Ka/Ks ratio is used to measure selective pressures acting on a gene, thus, being assumed as neutral when the net balance between deleterious and beneficial mutations (Ka/Ks = 1), positive or diversifying selection when Ka/Ks > 1, and negative or purifying selection when Ka/Ks < 1 [35].

2.4 Phylogenetic analyses

In order to reconstruct the mtDNA phylogenetic relationships among the three species analyzed here, and Chiroptera genera with previously published data, we used the amino acid sequences of all 13 PCGs of a total of 57 bat species with a complete mitogenome sequence, including *F. horrens*, *L. aurita*, *N. macrourus*, the 11 species used in the selective constraint analysis as mentioned above, and 43 additional species obtained from GenBank (Fig. 1). As outgroups for the analysis, we included the mitogenomes from

two other mammalian orders in the analysis as outgroup: *Ailurus fulgens* (Carnivora; MK886830) and *Mogera wogura* (Eulipotyphla; AB099482). Thus, after aligning the amino acid sequences of each gene individually using MAFFT v7.4 [36], with the alignment algorithm set to *Auto* and the JTT200 scoring matrix, the genes were concatenated into a single substitution matrix using Geneious Prime. Then, maximum likelihood (ML) and Bayesian inference (BI) trees were obtained by running RAxML v8.2 [37] and MrBayes v3.2 [38], respectively, as implemented in the CIPRES portal (https://www.phylo.org). The ML analysis was performed using the model PROTGAMMA and 1,000 replicates of rapid bootstrapping, and the BI trees were obtained using four simultaneous runs, each with four Markov chains (T = 0.2) extended through 20,000,000 generations, sampling every 2,000, and using a burn-in fraction of 25% of the trees.

Fig. 1. Phylogenetic relationships among Chiroptera genera using all mitogenome protein-coding genes. Majority-rule consensus tree based on Bayesian inference evidencing the phylogenetic relationships among the mitogenomes of *Furipterus horrens*. *Lonchorhina aurita*. *Natalus macrourus*. and 54 other bat genera. with one species each. indicating their respective family affiliations and GenBank accession numbers. The circles over the nodes indicate the following posterior probabilities: black. PP = 1.00; red 1.00 > PP ≥ 0.95; and white. 0.95 > PP ≥ 0.90. The triangles over the branches indicate the following bootstrap values obtained with the maximum likelihood approach: black. BS ≥ 90; red. 90 > BS ≥ 80; and white. 80 > BS ≥ 70.

3. Results and discussion

Among the analyzed species, *F. horrens* presented the most compact mitogenome, with a total length of 16,516 bp, followed by *L. aurita* and *N. macrourus*, with 16,697 bp and 16,668 bp, respectively. These lengths fall within the range of the complete bat mitogenomes deposited in GenBank as of up to date, which range from 16,175 bp in *Lasionycteris noctivagans* (MT774149) to 17,660 bp in *Submyotodon moupinensis* (MZ435948). As with all other members of Chiroptera, the mitogenomes of *F. horrens*, *L. aurita*, and *N. macrourus* contain the typical set of 37 genes, including 13 PCGs (ATP6, ATP8, COX1, COX2, COX3, CYTB, ND1, ND2, ND3, ND4, ND4L, ND5, and ND6), two ribosomal RNA genes [12S (rrnS) and 16S rRNA (rrnL)], 22 of transfer RNA (trnA, trnC, trnD, trnE, trnF, trnG, trnH, trnI, trnK, trnL1, trnL2, trnM, trnP, trnQ, trnR, trnS1, trnS2, trnT, trnV, trnW and trnY), and a control region (CR), which varied from 1,092 to 1,247 bp (Table 2).

Table 2. Mitochondrial genome of *Furipterus horrens* (ITV8911), *Lonchorhina aurita* (ITV8916), and *Natalus macrourus* (ITV8904). Arrangement and annotation. [a]H = heavy strand, and L = light strand.

Feature	Strand[a]	*Furipterus horrens*			*Lonchorhina aurita*			*Natalus macrourus*		
		Position (Length in bp)	Codons (start/stop)	overlap	Position (Length in bp)	Codons (start/stop)	overlap	Position (Length in bp)	Codons (start/stop)	overlap
trnF (GAA)	H	1-70 (70)	-	0	1-69 (69)	-	0	1-73 (73)	-	0
rrnS	H	71-1040 (970)	-	0	70-1044 (975)	-	0	74-1044 (971)	-	0
trnV (TAC)	H	1041-1107 (67)	-	0	1045-1113 (69)	-	0	1045-1112 (68)	-	0
rrnL	H	1108-2675 (1568)	-	0	1114-2685 (1572)	-	1	1113-2686 (1574)	-	1
trnL2(TAA)	H	2676-2750 (75)	-	2	2687-2761 (75)	-	2	2688-2762 (75)	-	2
ND1	H	2753-3709 (957)	ATG/TAA	-1	2764-3720 (957)	ATG/TAA	-1	2765-3721 (957)	ATG/TAA	-1
trnI (GAT)	H	3709-3777 (69)	-	-3	3720-3788 (69)	-	-3	3721-3789 (69)	-	-3
trnQ (TTG)	L	3775-3846 (72)	-	0	3786-3858 (73)	-	0	3787-3860 (74)	-	0
trnM (CAT)	H	3847-3915 (69)	-	0	3859-3927 (69)	-	0	3861-3929 (69)	-	0
ND2	H	3916-4959 (1044)	ATA/TAG	-2	3928-4974 (1047)	ATA/TAG	-2	3930-4973 (1044)	ATA/TAG	-2
trnW (TCA)	H	4958-5025 (68)	-	3	4973-5040 (68)	-	5	4972-5041 (70)	-	4
trnA (TGC)	L	5029-5097 (69)	-	1	5046-5114 (69)	-	0	5046-5116 (71)	-	1
trnN (GTT)	L	5099-5171 (73)	-	2	5115-5187 (73)	-	2	5193-5223 (31)	-	-1
trnC (GCA)	L	5205-5270 (66)	-	0	5221-5286 (66)	-	0	5223-5288 (66)	-	1
trnY (GTA)	L	5271-5336 (66)	-	1	5287-5353 (67)	-	1	5290-5355 (66)	-	1
COX1	H	5338-6882 (1545)	ATG/TAA	-3	5355-6899 (1545)	ATG/TAA	-3	5357-6913 (1557)	ATG/TAA	1
trnS2 (TGA)	L	6880-6948 (69)	-	7	6897-6965 (69)	-	7	6915-6983 (69)	-	7
trnD GTC	H	6956-7022 (67)	-	0	6973-7039 (67)	-	0	6991-7057 (67)	-	0
COX2	H	7023-7710 (688)	ATG/T	-3	7040-7723 (684)	ATG/TAA	3	7058-7741 (684)	ATG/TAA	3
trnK (TTT)	H	7708-7772 (65)	-	1	7727-7791 (65)	-	1	7745-7811 (67)	-	1
APT8	H	7774-7977 (204)	ATG/TAA	-43	7793-7996 (204)	ATG/TAA	-43	7813-8016 (204)	ATG/TAA	-43
ATP6	H	7935-8615 (681)	ATG/TAA	-1	7954-8634 (681)	ATG/TAA	-1	7974-8654 (681)	ATG/TAA	-1
COX3	H	8615-9398 (784)	ATG/T	0	8634-9419 (786)	ATG/TAG	-1	8654-9438 (785)	ATG/TA	-1
trnG (TCC)	H	9399-9467 (69)	-	0	9419-9487 (69)	-	-3	9438-9507 (70)	-	-3
ND3	H	9468-9815 (348)	ATA/TAA	0	9485-9835 (351)	ATT/TAA	0	9505-9855 (351)	ATT/TAA	0
trnR (TCG)	H	9816-9883 (68)	-	0	9836-9903 (68)	-	0	9856-9924 (69)	-	0
ND4L	H	9884-10180 (297)	ATG/TAA	-7	9904-10200 (297)	ATG/TAA	-7	9925-10221 (297)	ATG/TAA	20
ND4	H	10174-1551 (1378)	ATG/T	0	10194-11571 (1378)	ATG/T	0	10242-11622 (1381)	ATG/T	-30
trnH (GTG)	H	11552-11618 (67)	-	0	11572-11639 (68)	-	0	11593-11659 (67)	-	0
trnS1 (GCT)	H	11619-11677 (59)	-	1	11640-11698 (59)	-	1	11660-11718 (59)	-	1
trnL1 (TAG)	H	11679-11749 (71)	-	-9	11700-11769 (70)	-	-9	11720-11789 (70)	-	-9
ND5	H	11741-13567 (1827)	ATA/TAA	-17	11761-13587 (1827)	ATA/TAA	-17	11781-13610 (1830)	ATA/TAA	-17
ND6	L	13551-14075 (525)	ATG/TAA	3	13571-14098 (528)	ATG/TAA	0	13594-14121 (528)	ATG/TAA	0
trnE (TTC)	L	14079-14146 (68)	-	4	14099-14171 (69)	-	4	14122-14190 (69)	-	4
CYTB	H	14151-15290 (1140)	ATG/AGA	0	14172-15311 (1140)	ATG/AGA	0	14195-15334 (1140)	ATG/TAA	0
trnT (TGT)	H	15291-15357 (67)	-	0	15312-15381 (70)	-	-1	15335-15404 (70)	-	-1
trnP (TGG)	L	15358-15424 (67)	-	656	15381-15447 (67)	-	-40	15404-15470 (67)	-	682

For the three species, the genes were arranged similarly to all other bat species [39-41], with 29 genes (12 PCGs, the two rRNAs and 14 tRNAs) being located in the heavy (H) strand, while nine genes (ND6 and eight tRNAs) were observed in the light (L) strand (Table 2; Fig. 2). As the overall nucleotide composition depicted in Table 3, the GC contents for *F. horrens* (44.6%), *L. aurita* (40.5%), and *N. macrourus* (38.6%) is consistent with the range reported for other bats [41-42], also presenting an AT-skewed nucleotide composition in the PCGs, rRNAs and tRNAs, which is usually favored due to the lower energy cost to break AT bonds during replication and transcription processes in the mitogenome [43]. Furthermore, we observed a strand asymmetry for the three mitogenomes described here, with GC/AT deviations of −0.2421/0.1119 for *F. horrens*, −0.3234/0.0689 for *L. aurita*, and −0.3316/0.1339 for *N. macrourus*, indicating a higher content of pyrimidines in the L strand. The occurrence of asymmetries in mutational patterns may be attributed to replication and transcription mechanisms, which result in strand-specific biases and affect the likelihood of base substitutions between H and L strands, as each strand temporarily exists in a denatured (single-stranded) state, exposing the DNA to damage, and increasing its susceptibility to mutations [33,44].

Table 3. Nucleotide composition of *Furipterus horrens* (ITV8911), *Lonchorhina aurita* (ITV8916), and *Natalus macrourus* (ITV8904).

PCGs	31.8	28.9	12.6	26.7	58.5	41.5	0.088	-0.392	30.9	28.4	11.9	28.8	59.7	40.1	0.036	-0.409	34.0	27.0	11.6	27.5	61.5	38.5	0.105	-0.400
rrnS	35.4	24.4	18.1	22.1	59.6	42.5	0.232	-0.148	33.7	24.0	19.1	23.2	56.9	43.1	0.186	-0.114	36.9	23.1	17.1	23.0	59.9	40.2	0.232	-0.149
rrnL	38.2	21.5	17.5	22.8	61.0	39.0	0.252	-0.103	35.2	23.6	18.2	23.0	58.2	41.8	0.209	-0.129	39.0	19.8	16.6	24.5	63.5	36.4	0.228	-0.087
CR	31.7	26.9	15.8	25.6	57.3	42.7	0.105	-0.213	30.8	24.9	17.4	26.9	57.7	42.3	0.068	-0.144	33.1	29.4	14.8	22.7	55.8	44.2	0.188	-0.282

Fig. 2. Circular genome depiction of the mitochondrial genomes of *Furipterus horrens*, *Lonchorhina aurita*, and *Natalus macrourus*. Colored arrows pointing to the left and right represent the transcription regions of protein coding genes (blue), rRNA genes (red) and tRNA genes (purple) on the L and H strands, respectively.

The length and arrangement of the rRNA genes (rrnS and rrnL) in the analyzed mitochondrial genomes, also agreed with other bat species, ranging between 970-975 bp, and 1568-1574 bp, respectively. The rrnS gene was observed between trnF and trnV, the rrnL was located near the rrnS, between trnV and trnL2. In all three species, the two rRNA subunits displayed a positive AT-skew and a negative GC-skew. While the nucleotide composition of rrnS followed an A > C > T > G pattern, the nucleotide composition of rrnL varied among the analyzed species. *F. horrens* and *N. macrourus* exhibited an A > T > C > G composition, while *L. aurita* displays an A > C > T > G pattern.

All tRNA genes in the three species displayed the classic cloverleaf secondary structure, except for tRNA-S1, which differed from other tRNAs by lacking the DHU arm (Fig. S1-S3; Table S1). The absence of the DHU arm in trnS1 is a conserved trait among metazoans, and the secondary tRNA structures that we modelled agreed with those previously reported for other mammals [30,39]. The total length of tRNAs in *F. horrens*, *L. aurita*, and *N. macrourus* was between 1502 and 1523 bp, as shown in Table 2. The lengths of individual genes varied from 60 to 75 bp in trnS1 and trnL2, respectively, which also agrees with the lengths reported for other bat species in previous studies [39,41]. Due to the limited availability of detailed descriptions of tRNA secondary structures in Chiroptera, we provide a comparison among the three species described in this study with *Murina ussuriensis* and *Ectophylla alba*, which were previously analyzed by Yoon & Park [45] and Vivas-Toro et al. [41], respectively. Comparatively, the secondary structures of the tRNAs were very similar among species. However, slight variations in the length of the stem region in certain tRNAs were noted, as reported by Vivas-Toro et al. [41]. Except for trnR, which had a larger variation in length (S2

Table S2-S4), the length of the receptor stem is mostly consistent across all tRNAs. On the other hand, the length of the anticodon stem varied among species for three specific tRNAs: trnL2, trnQ, and trnS1. The TψC stem exhibited a greater variation in length, with variations observed in eight tRNAs: trnF, trnV, trnQ, trnC, trnR, trnH, trnL1, and trnT. Finally, the length of the DHU stem varied for six tRNAs, specifically trnN, trnC, trnS2, trnK, trnT, and trnS1 (trnS1 with the stem being absent as previously stated).

The mitogenomes of *F. horrens*, *L. aurita*, and *N. macrourus* showed minor differences in the start and stop codons of the PCGs. For *F. horrens*, only ND2, ND3 and ND5 have ATA as the start codon, whereas, as in the other two species, the remaining 10 PCGs utilize ATG. In the case of *L. aurita*, we observed ATA as alternative start codon in ND3 (Table 4). On the other hand, among the PCGs of *F. horrens*, eight PCGs utilize TAA as stop codon, while COX2 and CYTB use AGA, ND2 uses TAG, and COX3 and ND4 present T as an incomplete stop codon. For *L. aurita*, TAA is used as the stop codon for nine PCGs, while only CYTB uses AGA, ND2 and COX3 use TAG, and NAD4 presents T as incomplete stop codon (Table 4). Finally, for *N. macrourus*, we found TA and T as incomplete stop codons in COX3 and ND4, respectively, while ND2 presented TAA, and the 10 remaining PCGs with TAA as stop codon, as indicated in Table 4.

Table 4. Codon Usage of the 13 PCGs of the mitochondrial genomes of *Furipterus horrens. Lonchorhina aurita*, and *Natalus macrourus*.

Amino Acid	Codon	*Furipterus horrens*				*Lonchorhina aurita*				*Natalus macrourus*			
		Number	/1000	Fraction	RSCU	Number	/1000	Fraction	RSCU	Number	/1000	Fraction	RSCU
Ala (A)	GCG	4	1.05	0.02	0.08	4	1.05	0.02	0.08	7	1.84	0.04	0.16
	GCA	56	14.7	0.27	1.08	66	17.4	0.33	1.32	58	15.2	0.33	1.32
	GCT	58	15.2	0.28	1.12	71	18.7	0.36	1.44	44	11.6	0.25	1.00
	GCC	90	23.7	0.43	1.72	58	15.3	0.29	1.16	67	17.6	0.38	1.52
Cys (C)	TGT	17	4.47	0.53	1.06	9	2.37	0.28	0.56	7	1.84	0.28	0.56
	TGC	15	3.94	0.47	0.94	23	6.05	0.72	1.44	18	4.73	0.72	1.44
Asp (D)	GAT	27	7.10	0.47	0.94	46	12.1	0.53	1.06	53	13.9	0.55	1.10
	GAC	30	7.88	0.53	1.06	41	10.8	0.47	0.94	44	11.6	0.45	0.90
Glu (E)	GAG	27	7.10	0.30	0.60	50	13.1	0.41	0.82	39	10.2	0.35	0.70
	GAA	62	16.3	0.70	1.40	71	18.7	0.59	1.18	71	18.6	0.65	1.30
Phe (F)	TTT	57	15.0	0.38	0.76	89	23.4	0.41	0.82	79	20.8	0.42	0.84
	TTC	92	24.2	0.62	1.24	128	33.7	0.59	1.18	110	28.9	0.58	1.16
Gly (G)	GGG	13	3.42	0.10	0.40	17	4.47	0.11	0.44	24	6.30	0.15	0.60
	GGA	48	12.6	0.35	1.40	65	17.1	0.44	1.76	61	6.02	0.39	1.56
	GGT	27	7.10	0.20	0.80	34	8.94	0.23	0.92	38	9.98	0.24	0.96
	GGC	48	12.6	0.35	1.40	32	8.41	0.22	0.88	33	8.67	0.21	0.84
His (H)	CAT	75	19.7	0.49	0.98	60	15.8	0.47	0.94	83	21.8	0.49	0.98
	CAC	78	20.5	0.51	1.02	68	17.9	0.53	1.06	87	22.9	0.51	1.02
Ile (I)	ATT	110	28.9	0.49	0.98	115	30.2	0.55	1.10	123	32.3	0.61	1.22
	ATC	115	30.2	0.51	1.02	95	25.0	0.45	0.90	80	21.0	0.39	0.78
Lys (K)	AAG	20	5.26	0.16	0.32	20	5.26	0.19	0.38	19	4.99	0.18	0.36
	AAA	102	26.8	0.84	1.68	87	22.9	0.81	1.62	89	23.4	0.82	1.64
Leu (L)	TTG	21	5.52	0.05	0.30	31	8.15	0.06	0.36	28	7.35	0.06	0.36
	TTA	91	23.9	0.20	1.20	116	30.5	0.23	1.38	133	34.9	0.30	1.80
	CTG	42	11.0	0.09	0.54	23	6.05	0.05	0.30	20	5.25	0.05	0.30
	CTA	186	48.9	0.42	2.52	287	49.2	0.37	2.22	181	47.5	0.41	2.46
	CTT	48	12.6	0.11	0.66	64	16.8	0.13	0.78	44	11.6	0.10	0.60
	CTC	57	15.0	0.13	0.78	79	20.8	0.16	0.96	38	9.98	0.09	0.54
Met (M)	ATG	51	13.4	0.26	0.52	46	12.1	0.26	0.52	34	8.93	0.17	0.34
	ATA	145	38.1	0.74	1.48	129	33.9	0.74	1.48	163	42.8	0.83	1.66
Asn (N)	AAT	82	21.6	0.42	0.84	61	16.0	0.48	0.96	57	15.0	0.41	0.82
	AAC	111	29.2	0.58	1.16	66	17.4	0.52	1.04	82	21.5	0.59	1.18
Pro (P)	CCG	20	5.26	0.06	0.24	28	7.36	0.1	0.40	17	4.46	0.07	0.28
	CCA	128	33.6	0.41	1.64	96	25.2	0.33	1.32	103	27.1	0.42	1.68
	CCT	88	23.1	0.28	1.12	69	18.1	0.24	0.96	57	15.0	0.23	0.92
	CCC	80	21.0	0.25	1.00	95	25.0	0.33	1.32	70	18.4	0.28	1.12
Gln (Q)	CAG	55	14.5	0.39	0.78	48	12.6	0.31	0.62	46	12.1	0.26	0.52
	CAA	86	22.6	0.61	1.22	106	27.9	0.69	1.38	133	34.9	0.74	1.48
Arg (R)	CGG	7	1.84	0.11	0.44	1	0.26	0.02	0.08	3	0.79	0.07	0.28
	CGA	24	6.31	0.38	1.52	22	5.78	0.52	2.08	24	6.30	0.55	2.20
	CGT	14	3.42	0.21	0.84	9	2.37	0.21	0.84	5	1.31	0.11	0.44
	CGC	19	4.99	0.3	1.20	10	2.63	0.24	0.96	12	3.15	0.27	1.08
Ser (S)	AGT	17	4.47	0.05	0.30	20	5.26	0.05	0.30	11	2.89	0.03	0.18
	AGC	48	12.6	0.14	0.84	16	4.21	0.04	0.24	28	7.35	0.08	0.48
	TCG	24	6.31	0.07	0.42	27	7.10	0.07	0.42	30	7.88	0.09	0.54
	TCA	98	25.8	0.28	1.68	114	30.0	0.3	1.80	116	30.5	0.34	2.04
	TCT	87	22.9	0.25	1.50	88	23.1	0.23	1.38	65	17.1	0.19	1.14
	TCC	80	21.0	0.23	1.38	119	31.3	0.31	1.86	92	24.2	0.27	1.62
Thr (T)	ACG	22	5.78	0.06	0.24	25	6.57	0.09	0.36	25	6.57	0.08	0.32
	ACA	128	33.6	0.34	1.36	101	26.6	0.35	1.40	120	31.5	0.39	1.56
	ACT	101	26.5	0.27	1.08	66	17.4	0.23	0.92	54	14.2	0.18	0.72
	ACC	123	32.3	0.33	1.32	94	24.7	0.33	1.32	106	27.8	0.35	1.40
Val (V)	GTG	15	3.94	0.11	0.44	17	4.47	0.12	0.48	18	4.73	0.12	0.48
	GTA	63	16.6	0.47	1.88	60	15.8	0.42	1.68	60	15.8	0.4	1.60
	GTT	32	8.41	0.24	0.96	35	9.20	0.24	0.96	49	12.9	0.33	1.32
	GTC	25	6.57	0.19	0.76	31	8.15	0.22	0.88	23	6.04	0.15	0.60
Trp (W)	TGG	16	4.20	0.20	0.40	11	2.89	0.17	0.34	12	3.15	0.18	0.36
	TGA	65	17.1	0.80	1.60	53	13.9	0.83	1.66	55	14.4	0.82	1.64
Tyr (Y)	TAT	89	23.4	0.48	0.96	109	28.7	0.54	1.08	125	32.8	0.52	1.04
	TAC	96	25.2	0.52	1.04	93	24.5	0.46	0.92	115	30.2	0.48	0.96
Stop (*)	AGG	18	4.73	0.12	0.48	2	0.53	0.01	0.04	2	0.53	0.01	0.04
	AGA	14	3.68	0.09	0.36	8	2.10	0.04	0.16	2	0.53	0.01	0.04
	TAG	51	13.4	0.34	1.36	79	20.8	0.42	1.68	94	24.7	0.43	1.72
	TAA	68	17.9	0.45	1.80	101	26.6	0.53	2.12	122	32.0	0.55	2.20

Regarding the codon usage bias, it is widely recognized that vertebrates tend to favor particular synonymous codons, which can differ depending on the species [46]. In order to assess codon usage bias within the studied species, we utilized the RSCU method, which involves comparing the observed frequency of a synonymous codon to the expected frequency of that same codon when all codons are used equally for a given amino acid. As previously observed for *E. alba* [41], we noticed that the PCGs of *F. horrens*, *L. aurita*, and *N. macrourus* utilize multiple codons to encode each amino acid, following the pattern already documented, although the codon usage frequency differed among the three studied species, as indicated in Table 4. Codons ending in adenine are preferred, while codons ending in guanine were the least used [39,41]. The most frequently occurring codon among the three species was CTA, which corresponds to the amino acid Leucine (Leu), appearing 186 times in the PCGs of *F. horrens*, 287 of *L. aurita*, and 181 of *N. macrourus* (Table 4). In contrast, the codon CGG for Arginine (Arg) is the least frequently used, being observed seven times in *F. horrens*, once in *L. aurita*, and three times in *N. macrourus* (Table 4).

The ratio of synonymous (Ks) and non-synonymous (Ka) substitutions is a straightforward way to measure the selection pressure on a gene. This ratio indicates neutrality when Ka/Ks = 1, negative selection when Ka/Ks < 1, and positive selection when Ka/Ks > 1, thus aiding to understand evolutionary gene selections [47-48]. Our analyses revealed that all PCGs of *F. horrens, L. aurita* and *N. macrourus* have Ka/Ks < 1, intense purifying selection. Among the other protein-coding genes (PCGs), the ATP8 gene showed the highest value for the Ka/Ks ratio (0,618 ± 0,123), indicating that it experienced less affected by selective pressure (Fig. 3).

Fig. 3. Selective pressure analysis in the protein coding genes of *Furipterus horrens*, *Lonchorhina aurita*, and *Natalus macrourus*. Ka/Ks ratio for each protein coding gene.

Furthermore, the analyzed mitogenomes have overlapping regions between genes, being similar to what has been observed for other mammals (e.g., [49-52]). As previously reported in other bat mitogenome descriptions, we noticed three long overlapping regions in all three species (located between ATP8 and ATP6, ND5 and ND6, and ND4L and ND4). Additionally, we observed shorter overlaps ranging from 1-3 bp, which are detailed in Table 2. *Furipterus horrens*, *L. aurita*, and *N. macrourus* have slightly different CR lengths of 1092 bp, 1252 bp, and 1198 bp, respectively.

Apart from genes, the organellar genome of animals is also composed of non-coding regions, including the origin of replication (OriL), the D-loop complex (or control region – CR), and a few intergenic spacers, playing a crucial role in the replication and maintenance of the mitochondrial genome [49]. As anticipated, these regions were present in all three species covered in this study. The intergenic spacers of the three species analyzed have total lengths of 25, 27, and 29 bp in *F. horrens*, *L. aurita* and *N. macrourus*, respectively, falling within the range of 18-43 bp reported for other bats species in the same taxonomic order, as indicated by Meganathan et al. [39]. According to Seutin et al. [53], OriL is found in the WANCY region between the genes for trnA and trnC in vertebrates. Within Chiroptera, OriL ranges from 31-34 bp, being also identified in three species we analyzed (32 bp in both *F. horrens* and *L. aurita*, and 31 bp in *N. macrourus*).

In bats, the CR typically has a GC content slightly below the AT content, as also observed in all three of the analyzed mitochondrial genomes (*F. horrens* with 42.7%, *L. aurita* with 42.3%, and *N. macrourus* with 44.2%). Considering the repetitive portions of the mitogenomes, we observed eight, 13, and 10 microsatellites for *F. horrens, L. aurita*, and *N. macrourus*, respectively. Most of these microsatellites have repeated sequences of TA or AT dinucleotides (Tables S2-S4). Furthermore, the three functional domains (extended terminal associated sequences [ETAS], core, and conserved sequence block [CSB]) present in other bats

and most mammals [54-55,41] were also observed in the CR of *F. horrens*, *L. aurita*, and *N. macrourus*.

The mitogenomes were grouped into two major clades, with the first including Megadermatidae, Hipposideridae, Rhinolophidae and Pteropodidae, and the second being composed of Emballonuridae, Mystacinidae, Noctilionidae, Furipteridae, Mormoopidae, Phyllostomidae, Natalidae, Molossidae and Vespertilionidae (Fig. 1). In general, the relationships among and within families were well resolved, with most of the groups presenting strong support values (Fig. 1). The most notable exception occurred between Molossidae and Vespertilionidae, which were recovered as paraphyletic, although with relevant support only in the Bayesian inference, with *Tadarida teniotis* (Molossidae) and *Miniopterus fulilginosus* (Vespertilionidae) appearing as sister lineages (Fig. 1), with a conflicting pattern involving both families being previously observed in the topology based on transcriptome data presented by Lei & Dong (2016). Considering the phylogenetic position of the three new mitogenomes, *F. horrens* (Furipteridae) was recovered as sister to *Noctilio leporinus* (Noctilionidae) with strong support, *L. aurita* and *Tonatia bidens* shared a node with moderate support within Phyllostomidae, and *N. macrourus* (Natalidae) appeared as sister to the group formed by Molossidae and Vespertilionidae species (Fig. 1).

4. Conclusion

In this study, we sequenced and analyzed the complete mitochondrial genome of three endangered cave-bat species, *F. horrens, L. aurita*, and *N. marourus*. We studied in detail these organellar genomes, presenting their nucleotide composition, bias and codon usage, the secondary structure of tRNAs, and CR characteristics of these species. The assembly and detailed analysis of the complete mitochondrial genome can be helpful for phylogenetic studies, helping to identify species in monitoring approaches such as those based on eDNA sampling.

The characterization of genetic resources represents a step forward in efforts to conserve bat species, especially endangered ones, as they allow measuring and managing the genetic diversity of populations. Thus, the comprehensive mitogenome analysis and the several genetic markers we described for the three mitogenomes will be useful for future studies on the evolutionary patterns of the populations of the three species.

Acknowledgments

This work was funded by Vale (Projeto Diversidade Biológica de Cavernas, R100603.CD.0X). Guilherme Oliveira is a CNPq (Conselho Nacional de Desenvolvimento Científico) fellow (307479/2016-1). The authors would like to thank the laboratory technician Manoel Lopes.

Conflict of interest

The authors declare no conflict of interest.

Data availability

All data is available at NCBI's GenBank.

References

1. Wilson DE, Mittermeier RA. Handbook of the Mammals of the World - Vol. 9, Bats. Lynx Editions, Barcelona. 2019; pp. 1008. ISBN: 978-84-16728-19-0.
2. Ferreiras RL, Martins RP. Trophic structure and natural history of bat guano invertebrate communities, with special reference to Brazilian caves. Tropical Zoology, 1999. 12:231-252.

3. Kunz HK. De Torrez EB, Bauer DM, Labova TA, Fleming TH. Ecossystem services provided by bats. Ann New York Acad Sci, 2011; 1223: 1-28.
4. Kunz TH. RoostIng ecology of bats, In: Kunz, T. H. Ecology of bats. New York: Plenum Press, 1982. pp. 1-55.
5. Rocha PA. Quiropterofauna cavernícola: composição, estrutura de comunidade, distribuição geográfica. Tese de doutorado em Ciências Biológicas (Zoologia), Programa de Pós-graduação em Ciências Biológicas, 2013; Universidade Federal da Paraíba. João Pessoa. Paraíba
6. Pavan AC, Tavares VC. Pteronotus gymnonotus (Chiroptera: Mormoopidae). Mammalian Species, 2020, v. 52, pp. 40-48.
7. Auler AS, Farrant AR. A brief introduction to karst and caves in Brazil. Proceedings of University of Bristol Spelaeological Society, 1996; 20(3) 187-200.
8. Centro Nacional de Pesquisa e Conservação de Cavernas (CECAV). Cadastro Nacional de Informações Espeleológicas – CANIE, 2019. Available from: https://www.gov.br/icmbio/pt-br/assuntos/centros-de-pesquisa/cecav.
9. Bernard E, Aguiar LMS, Machado RB. Discovering the Brazilian bat fauna: a task for two centuries? Mammal Review, 2011; 41:23–39.
10. Paglia AP, Fonseca GAB, Rylands AB, Herrmann G, Aguiar LMS, Chiarello AG, Leite YLR, Costa LP, Siciliano S, Kierulff MCM, Mendes SL, Tavares VC, Mittermeier RA, Patton JL. Lista Anotada dos Mamiferos do Brasil. 2° Edição, Conservation International, Arlington, VA, 2011; 4:76.
11. Tavares VC, Palmuti CF, Gregorin R, Dornas TT. Morcegos. In: Martins FD, Castilho A, Rolim S. Fauna da floresta nacional de Carajás: estudos sobre vertebrados terrestres. Nitro Editorial, 2012.
12. Garbino GST, Gregorin R, Lima-Loureiro IPL, Moras LM, Moratelli R, Nogueira MR, Pavan AC, Tavares VC, Peracchi AL. Updated checklist of Brazilian bats. Comitê da Lista de Morcegos do Brasil: Sociedade Brasileira para o Estudo de Quirópteros, 2020.
13. Simmons NB, Cirranello AL. Bat Species of the World: A taxonomic and geographic database. New York: American Museum of Natural History, 2022. Available from: www.batnames.org.
14. Holsinger IR, Culver DC. The invertcbrate cave fauna of Virginia and a part of eastern Tennesscc: zoogeography and ecology. Brimleyana, 1988; 11: 164.
15. Trajano E. Cave faunas in the Atlantic tropical rain forest: composition, ecology, and conservation. Biotropica, 2000; 32: 882–893.

16. Di Lorenzo T, Di Marzio WD, Spigoli D, Baratti M, Messana G, Cannicci S, Galassi D M. Metabolic rates of a hypogean and an epigean species of copepod in an alluvial aquifer. Freshwater Biology, 2015; 60: 426–435.
17. Reboleira ASPS. Abrantes NA, Orami P, Gonçalves F. Acute toxicity of copper sulfate and potassium dichromate on Stygobiont Proasellus: general aspects of groundwater ecotoxicology and future perspectives. Water, Air & Soil Pollution, 2013; 224: 1550.
18. Frick WF, Kingston T, Flanders J. A review of the major threats and challenges to global bat conservation. Annals of the New York Academy of Sciences, 2019; 1469: 5–25.
19. Barquez R, Diaz M. Artibeus planirostris. In: IUCN 2013. IUCN Red List of Threatened Species. Version 2013.2; 2009. Available from: http://www.iucnredlist.org.
20. Cuvier, F. Description d'un nouveau genre de chauve-souris sous le nom de Furie. G. Dufour. Mémoires du Muséum d'histoire naturelle, 1828; 16: 150.
21. Tomes, R. F. On a new genus and species of leaf-nosed bats in the museum at Fort Pitt. Proc. Zool. Soc. London, 1863:81-84.
22. Gervais, P. Deuxième mémoire. Documents zoologiques pour servir à la monographie des Chéiroptères sud-américains. In: (P. Gervais, ed.) Mammifères. In: (F. de Castelnau, ed.) Animaux nouveaux ou rares recueillis pendant l'expédition dans les parties centrales de l'Amérique du Sud, de Rio de Janeiro à Lima, et de Lima au Para. P. Bertrand, Paris, France. 1856. pp. 25–88.
23. DÁvalos L, Tejedor A. Natalus espiritosantensis. In: The IUCN Red List of Threatened Species. Version 2014.2, 2008.
24. Leys R, Watts CH, Cooper SJ, Humphreys WF. Evolution of subterranean diving beetles (Coleoptera: Dytiscidae: Hydroporini, Bidessini) in the arid zone of Australia. International Journal of Organic Evolution, 2003; 57: 2819- 2834.
25. McCormack JE, Hird SM, Zellmer AJ, Carstens BC, Brumfield RT. Applications of next-generation sequencing to phylogeography and phylogenetics. Molecular Phylogenetics and Evolution, 2013; 66: 526-538.
26. Hollingsworth PM, Li D, VanderBank M, Twyford AD. Telling plant species apart with DNA: from barcodes to genomes. Philosophical Transactions of the Royal Society, Series B, 2016; 371.
27. Schubert M, Lindgreen S, Orlando L. AdapterRemoval v2: rapid adapter trimming, identification, and read merging. BMC Res notes, 2016. doi:10.1186/s13104-016-1900-2
28. Dierckxsens N, Mardulyn P, Smits G. NOVOPlasty: de novo assembly of organelle genomes from whole genome data. Nucleic Acids Res., 2017; 45(4): 18.

29. Bernt M, Donath A, Jühling F, Externbrink F, Florentz C, Fritzsch G, Pütz J, Middendorf M, Stadler PF. MITOS: Improved de novo metazoan mitochondrial genome annotation. Mol. Phyl. Evol., 2013; 69: 313–319.
30. Jühling F, Pütz J, Bernt M, Donath A, Middendorf M, Florentz C, Stadler PF. Improved systematic tRNA gene annotation allows new insights into the evolution of mitochondrial tRNA structures and into the mechanisms of mitochondrial genome rearrangements. Nucleic Acids Res., 2012; 40(7): 2833–2845.
31. Kerpedjiev P, Hammer S, Hofacker IL. Forna (force-directed RNA): Simple and effective online RNA secondary structure diagrams. Bioinformatics, 2015; 31(20): 3377–3379.
32. Bikandi J, San Mill´an R, Rementeria A, Garaizar J. In silico analysis of complete bacterial genomes: PCR, AFLP-PCR, and endonuclease restriction. Bioinformatics, 2004; 20: 798–799.
33. Perna N, Kocher T. Patterns of nucleotide composition at fourfold degenerate sites of animal mitochondrial genomes. Journal of Molecular Evolution, 1995; 41(3).
34. Tamura K, Steche G, Kumar S. MEGA11: Molecular Evolutionary Genetics Analysis Version 11. Molecular Biology and Evolution, 2021; 38(7): 3022–3027.
35. Wang D, Wan H, Zhang S, Yu J. γ-MYN: a new algorithm for estimating Ka and Ks with consideration of variable substitution rates. Biol. Direct, 2009; 4: 20.
36. Katoh K, Standley DM. MAFFT multiple sequence alignment software version 7: improvements in performance and usability. Molecular Biology and Evolution, 2013; 30(4): 772-780.
37. Stamatakis A. RAxML version 8: a tool for phylogenetic analysis and post-analysis of large phylogenies. Bioinformatics, 2014; 30(9): 1312-1313.
38. Ronquist F, Teslenko M, Van der Mark P, Ayres DL, Darling A, Höhna S, Larget B, Liu L, Suchard MA, Huelsenbeck JP. MrBayes 3.2: efficient Bayesian phylogenetic inference and model choice across a large model space. Systematic Biology, 2012; 61(3): 539-542.
39. Meganathan PR, Pagan HJT, McCulloch ES, Stevens RD, Ray DA. Complete mitochondrial genome sequences of three bats species and whole genome mitochondrial analyses reveal patterns of codon bias and lend support to a basal split in Chiroptera. Gene, 2012; 492(1): 121-129.
40. Wang Z, Wang N, Liang H. Knowledge sharing, intellectual capital and firm performance. Management Decision, 2014; 52(2): 230–258.
41. Vivas-Toro I, Ortega J, Baeza JA. The complete mitochondrial genome of the Honduran white bat Ectophylla alba (Allen 1982) (Chiroptera: Phyllostomidae). Gene, 2021; 802.

42. Botero-Castro F, Tilak M, Justy F, Catzeflis F, Delsuc F, Douzery EJP. Next-generation sequencing and phylogenetic signal of complete mitochondrial genomes for resolving the evolutionary history of leaf-nosed bats (Phyllostomidae). Mol. Phylogenet. Evol., 2013; 69: 728–739.
43. Silvestre D, Dowton M, Arias MC. The mitochondrial genome of the stingless bee Melipona bicolor (Hymenoptera, Apidae, Meliponini): sequence, gene organization and a unique tRNA translocation event conserved across the tribe Meliponini. Genetics and Molecular Biology, 2008; 31(2): 451–460.
44. Hassanin A, Leger N, Deutsch J. Evidence for multiple reversals of asymmetric mutational constraints during the evolution of the mitochondrial genome of Metazoa, and consequences for phylogenetic inferences. Syst. Biol. 54, 2005; 277–298.
45. Yoon KB, Park YC. Secondary structure and feature of mitochondrial tRNA genes of the Ussurian tube-nosed bat Murina ussuriensis (Chiroptera: Vespertillionidae). Genomics Data, 2015; 5: 213-217.
46. Grantham R, Gautier C, Gouy M, Mercier R, Pavé A. Codon catalog usage and the genome hypothesis. Nucleic Acids Research, 1980; 8(1): 197–197.
47. Tiffin P, Hahn M. Coding Sequence Divergence Between Two Closely Related Plant Species: Arabidopsis thaliana and Brassica rapa ssp. Pekinensis. J Mol Evol, 2002; 54: 746–753.
48. Yang Z, Nielsen R. Estimating Synonymous and Nonsynonymous Substitution Rates Under Realistic Evolutionary Models. Molecular Biology and Evolution, 2000; 17(1): 32–43.
49. Fernandez-Silva P, Enriquez JA, Montoya J. Replication and transcription of mammalian mitochondrial DNA. Exp. Physiol., 2003; 88: 41-56.
50. Ki JS, Hwang DS, Park TJ, Han SH, Lee JS. A comparative analysis of the complete mitochondrial genome of the Eurasian otter Lutra lutra (Carnivora; Mustelidae). Mol. Biol. Rep., 2009; 37(4): 1943-1955.
51. Tan S, Xu JT, Zou FD, Peng QK, Peng R. The complete mitochondrial genome of leopard cat, Prionailurus bengalensis chinensis (acrnivora: Felidae). Mitochondrial DNA, 2016; 27(5): 3073-3074.
52. Pang X. Complete mitochondrial genome of Tlacuatzin canescens (Grayish Mouse Opossum). Mitochondrial DNA A DNA Mapp Seq Anal., 2017; 28(2): 283-284.
53. Seutin G, Lang BF, Mindell DP, Morais R. Evolution of the WANCY region in amniote mitochondrial DNA. Mol Biol Evol. 1994 May;11(3):329-40.

54. Wilkinson GS. Fleming TH. Migration and evolution of lesser long-nosed bats Leptonycteris curasoae, inferred from mitochondrial DNA. Mol. Ecol., 1996; 5: 329–339.
55. Pumo DE, Finamore PS, Franck WR, Phillips CJ, Tarzami S, Balzarano D. Complete Mitochondrial Genome of a Neotropical Fruit Bat, Artibeus jamaicensis, and a New Hypothesis of Relationships of Bats to Other Eutherian Mammals. J. Mol. Evol., 1998; 47: 709–717.
56. Lei M, Dong D. Phylogenomic analyses of bat subordinal relationships based on transcriptome data. Scientific Report, 2016; 6-27726.

Supporting information

Fig. S1. Secondary structure of the 22 tRNAs in the mitochondrial genome of *Furipterus horrens*.

Fig. S2. Secondary structure of the 22 tRNAs in the mitochondrial genome of *Lonchorhina aurita*.

Fig. S3. Secondary structure of the 22 tRNAs in the mitochondrial genome of *Natalus macrourus*.

Table S1. Comparison of the length of the four stems that conform the secondary structure of the tRNAs of *Furipterus horrens*, *Lonchorhina aurita,* and *Natalus macrourus*.

Table S2. Microsatellites sequences found in the CR of the mitochondrial genome of *Furipterus horrens*.

Table S3. Microsatellites sequences found in the CR of the mitochondrial genome of *Lonchorhina aurita*.

Table S4. Microsatellites sequences found in the CR of the mitochondrial genome of *Natalus macrourus*.

Figure 1

Click here to access/download;Figure;Fig_1.eps

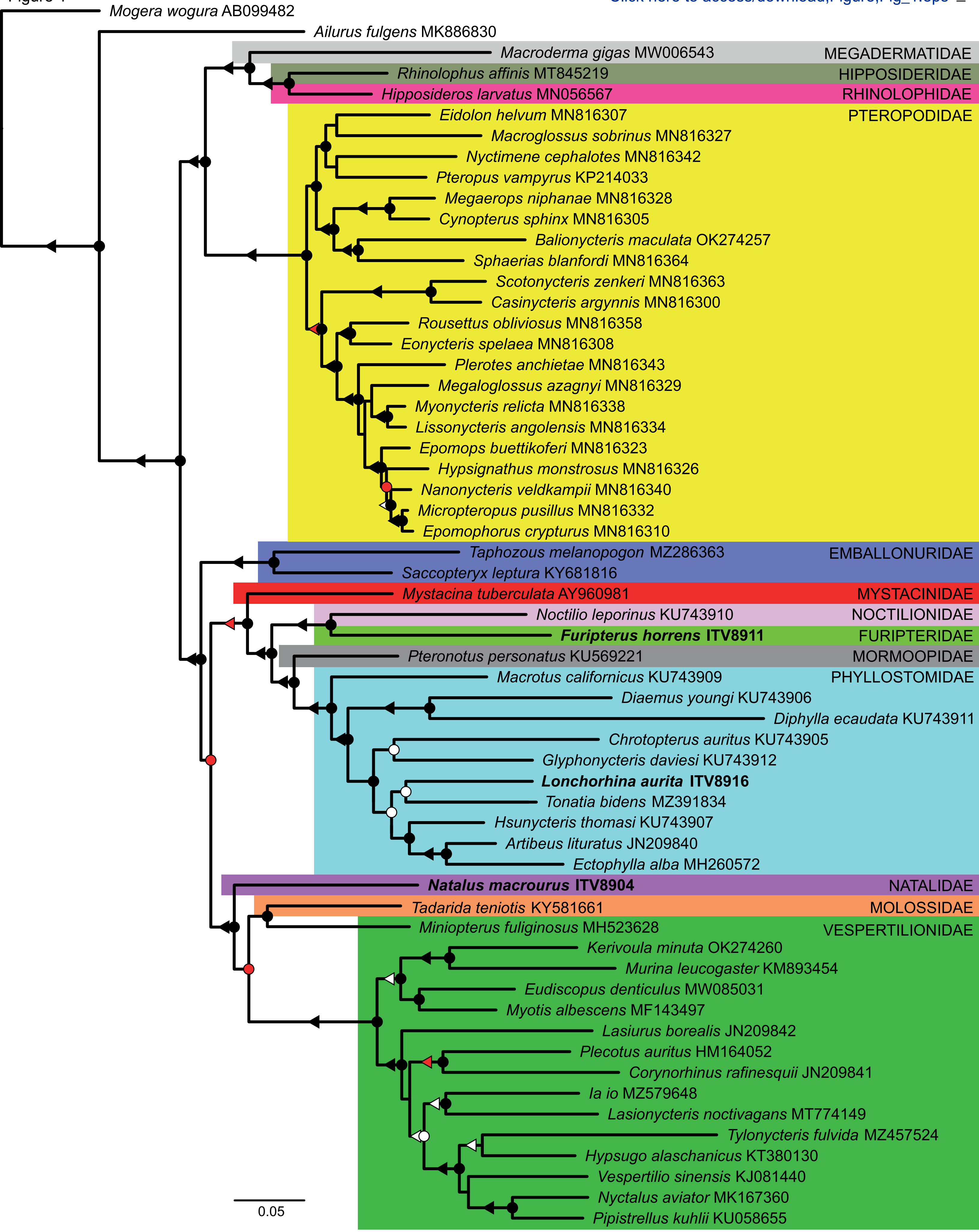

Figure 2

Click here to access/download;Figure;Fig_2.jpg

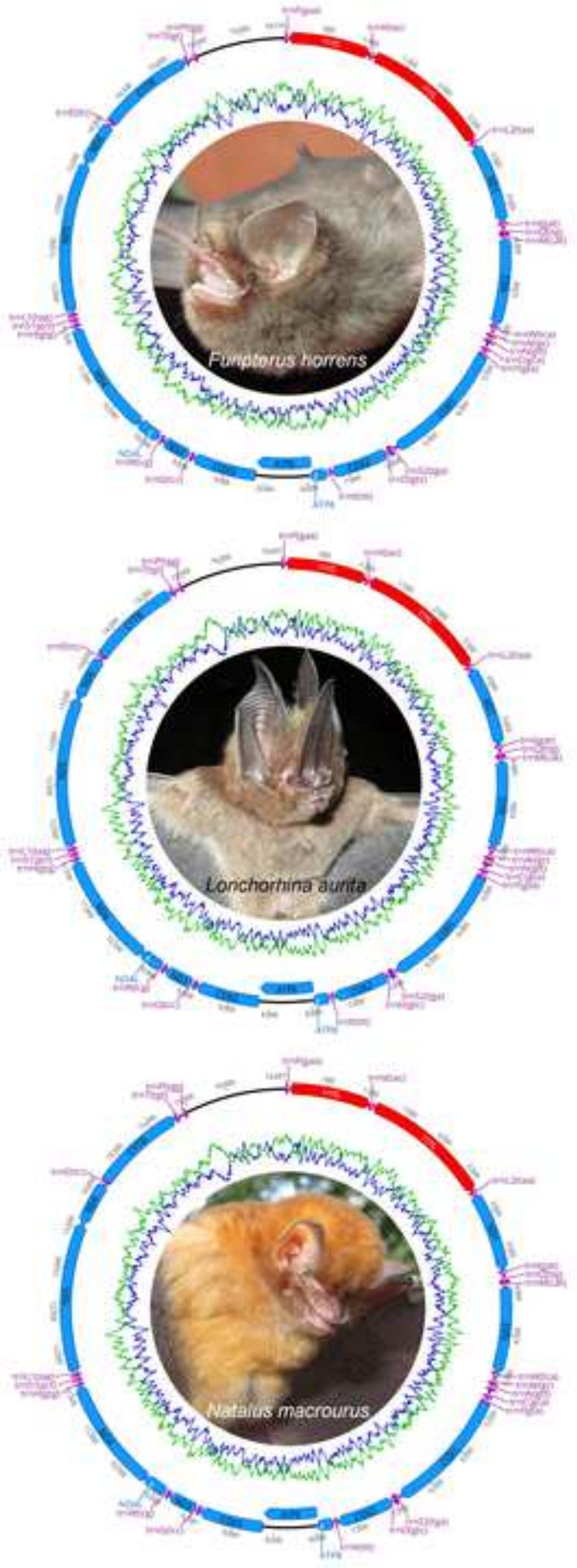

Figure 3

Click here to access/download;Figure;Fig_3.eps

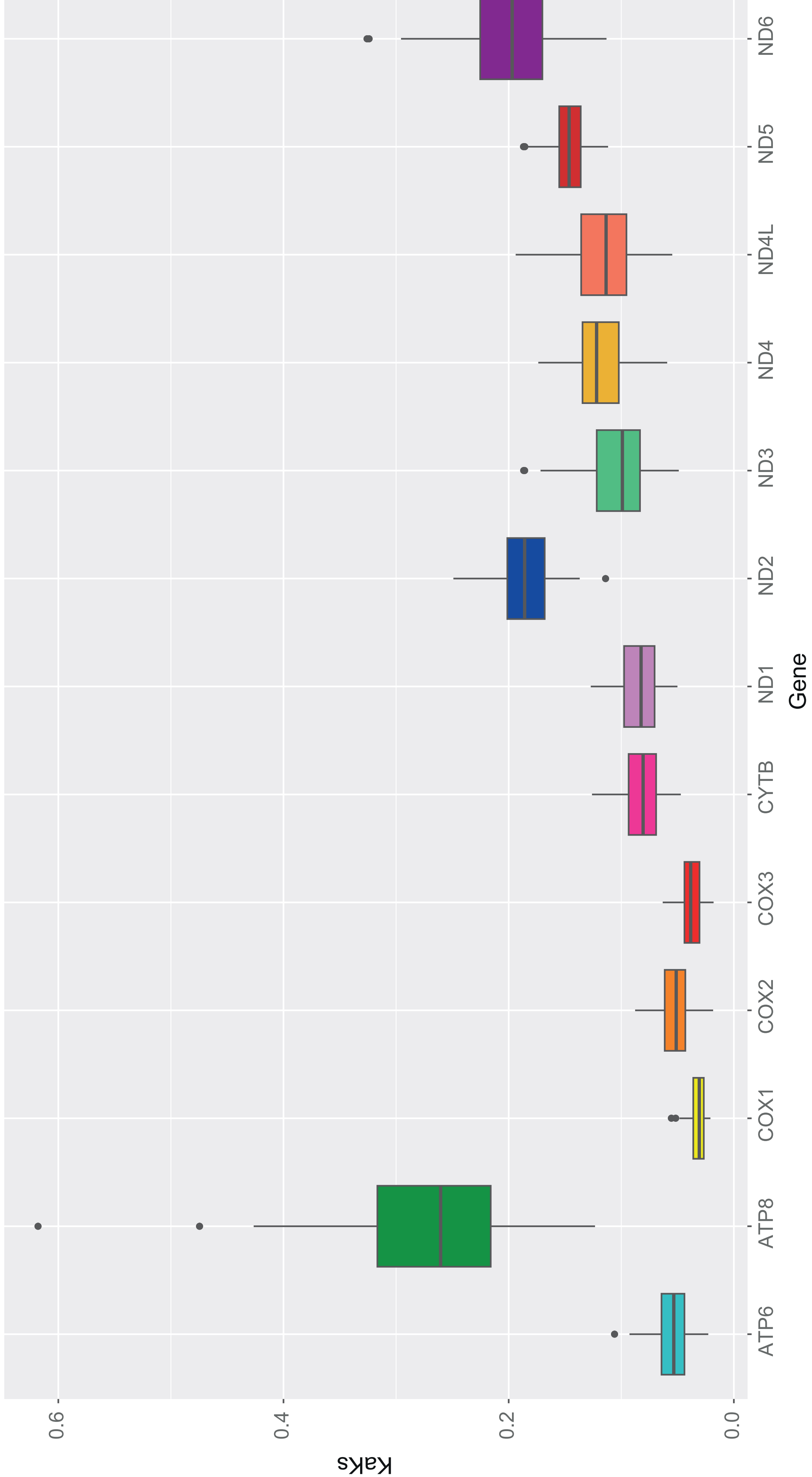

Supplementary Figure S1

Click here to access/download
Supporting Information
Fig_S1_tRNA_fig_Fhorrens.pdf

Supplementary Figure S2

Click here to access/download
Supporting Information
Fig_S2_tRNA_fig_Laurita.pdf

Supplementary Figure S3

Click here to access/download
Supporting Information
Fig_S3_tRNA_fig_Nmacrourus.pdf

Supplementary Tables

Click here to access/download
Supporting Information
Molina_et_al_ChiropteraMitogenome_SupTables_vf.docx
