# Supplementary material for: Complete mitochondrial genomes of three vulnerable cave bat species and their phylogenetic relationships within the order Chiroptera
Source: PLoS One. 2024 Aug 22;19(8):e0308741. doi: 10.1371/journal.pone.0308741 (PMC11340975; doi:10.1371/journal.pone.0308741)
Supplement: Attachment — Submitted filename: Molina_et_al_Response_to_reviewers.docx [file pone.0308741.s010.docx]

To

**Dr. Pankaj Bhardwaj**

**PLoS One**

**Academic Editor**

Dear Editor,

We are grateful for the reviews we received, which undoubtedly will contribute to enhancing our work. We have revised the manuscript thoroughly and the text was modified according to the comments of the reviewers. All featured comments are listed and answered below.

**REVIEWER #1**

*The title should be improved. This is not only about new sequence but also the thorough analyses of all known sequences available on this order. So, the phylogeny implication should add in the title.*

*"Complete mitochondrial genome of three vulnerable cave bat species with phylogenetic implications for the order Chiroptera"*

- We have changed the title to “Complete mitochondrial genomes of three vulnerable cave bat species and their phylogenetic relationships within the order Chiroptera”.

*1. Kindly discuss about the genome arrangement in the complete mitochondrial genome. As the mitogenomes of some animal groups exhibit novel rearrangements in gene. Is there any such rare rearrangements exist so far in this order?*

- We have included the suggested discussion in the beginning of the second paragraph of the “Results and discussion” section.

*2. Page 15 [Line 10] we observed eight, 13, and 10 microsatellites*

*13? Please compare with Table S4.*

- We have corrected this information in the text and the respective Supplementary Tables.

*3. Please provide a table with species name, accession number, genome size and reference.*

- We have included the GenBank accession numbers and mitogenome sizes in the Table 1.

________________________________________________________________

**REVIEWER #2**

*-Table 1: It is recommended to use a sampling map instead of a table.*

- We have included a map with the sampling points in the Figure 1 (former Figure 2), alongside the mitogenome depictions of the three species.

*-Table 2: The overlap between trnP and trnF is 656 bp? Please check and verify all data.*

- We have checked the data and corrected this information in Table 2.

*-Table 3: The table is incomplete.*

- We have corrected Table 3 putting the headings that were missing.

*-Fig. 1: What does “la io” mean.*

- *Ia io* (great evening bat) is a species from southeastern Asia that is included in the family Vespertilionidae.

*-Fig. 2: The resolution of Figure 2 can be improved.*

- As it contains photos of the bats, Figure 2 is a bitmap image. Due to file size and format restrictions, its resolution was severely lowered in the upload process to the submission platform. We agree that the resolution as reviewed was inappropriate, and we will provide the figure with a proper resolution once we can upload the file.

*-Line 1: Should be “genomes…”, not “genome…”.*

- We have modified the text accordingly.

*-Line 133: Is it GC skew or GC-skew?*

- We have modified the text accordingly, using GC-skew in all mentions.

*-Line 159: Is the phylogenetic tree constructed using 13 amino acid sequences or 13 PCGs?* *According to line 177, the tree was constructed using the PCGs.*

- The phylogenetic analyses were performed with the amino acid sequences of the 13 PCGs. We have modified the text to clarify the points mentioning the matrix used in the phylogenetic reconstructions.

*-Lines 178-179: Replace “.” with “,”.*

- We have modified the text accordingly.

________________________________________________________________

**REVIEWER #3**

*1. The results of the phylogenetic analysis should be shown in "Abstract".*

- As requested, we have included the part of the phylogenetic analysis in the abstract.

*2. The table header is missed in "Table 3".*

- We have corrected Table 3 putting the headings that were missing.

*3. In the list of references, the format of the journal names is inconsistent. Please check and make revisions as required by the journal format.*

- We have checked and corrected the references, according to the journal format.

*4. The English language of this manuscript needs to be revised for clarity. For example:*

*(1) Line 28, Suggested reword "and each comprises......"*

*(2) Line 96, Suggested reword "......Finally, we assessed the"*

*(3) Line 98, Suggested reword "on the amino acid supermatrix of the 13 PCGs."*

*(4) Line 127, Suggested reword "checked and curated in Geneious. The tRNAs ......"*

*(5) Line 171, Suggested reword "...... the BI tree was"*

*(6) Line 178, Suggested reword "...... of Furipterus horrens, "*

*(7) Line 179, Suggested reword "Lonchorhina aurita, Natalus macrourus, and 54 other bat genera with one species each, "*

*(8) Line 181, Suggested reword "......black PP = 1.00; red "*

*(9) Line 182, Suggested reword "......and white 0.95 > PP ≥ 0.90. The triangles ...... "*

*(10) Line 183, Suggested reword "...... approach: black"*

*(11) Line 324, Suggested reword "The Chiroptera was grouped ......"*

- We have carefully revised the manuscript and corrected all issues related to the English language, including all points mentioned by the reviewer.

In addition to the corrections indicated by the reviewers, we also included other modifications, which are also highlighted in the text, aiming to improve the manuscript.

Thank you very much for your kind attention.

Sincerely yours,

**Santelmo Vasconcelos**

Instituto Tecnológico Vale

Rua Boaventura da Silva 955

66055-090 Belém, Pará, Brazil

Phone: +55 91 3213 5400

santelmo.vasconcelos@itv.org
